# *Clostridioides difficile* Infections among Pediatric Patients Hospitalized at an Oncology Department of a Tertiary Hospital in Poland

**DOI:** 10.3390/medicina59081363

**Published:** 2023-07-25

**Authors:** Ewelina Lemiech-Mirowska, Ewelina Gaszyńska, Aleksandra Sierocka, Zofia Kiersnowska, Michał Marczak

**Affiliations:** 1 Department of Management and Logistics in Healthcare, Medical University of Lodz, 90-419 Lodz, Poland; adreslewska@wp.pl (A.S.); zofiakiersnowska.p@gmail.com (Z.K.); michal.marczak@umed.lodz.pl (M.M.); 2 Department of Nutrition and Epidemiology, Medical University of Lodz, 90-419 Lodz, Poland; ewelina.gaszynska@umed.lodz.pl

**Keywords:** *Clostridioides difficile* infection, clinical condition parameters, colonisation, paediatric patients, cancers, diarrhoea

## Abstract

*Background and Objectives*: Gastrointestinal tract infections caused by *Clostridioides difficile* bacteria are diagnosed in pediatric patients with increasing frequency. Children treated at pediatric units are a group of patients at high risk of this infection; therefore, appropriate differential diagnostics and an individual approach to every case are of particular importance. The goal of the study was to assess the clinical parameters of patients with a confirmed CD infection and colonization. *Materials and Methods*: Every positive case was subjected to a retrospective analysis based on medical history and an infection notification note. *Results*: Positive results were obtained for 30 patients, among whom the results of 18 patients were considered to justify the diagnosis of an infection. In the remaining patients, treatment was not initiated in only three cases. Cases were detected where treatment was initiated despite the lack of sufficient clinical evidence. *Conclusions*: This study demonstrates that there are many factors that result in a high risk of the occurrence of CDI in oncology patients, such as antibiotic therapy, multiple hospitalizations, and myelosuppression.

## 1. Introduction

Infections caused by toxigenic strains of *Clostridioides difficile* (CD) constitute a growing public health challenge, both clinically and economically [1]. Pediatric patients treated at oncology units are a group of patients at an especially high risk of a *Clostridioides difficile* infection (CDI) [2]. On the one hand, one has to remember the high level of CD colonization in children up to 2 years of age and the need to exclude other infectious and non-infectious factors before the final diagnosis [3]. On the other hand, oncological treatment is related to a high risk of complications, extended hospitalizations, the use of aggressive treatments impacting the entire immune system, and thus a significant probability of infections that require the introduction of protracted antibiotic therapy [4]. The basis of oncological treatment is the administration of cytostatic drugs, which inhibit and disrupt the cellular division process, resulting in the apoptosis of rapidly dividing cancer cells. This also affects healthy host cells that undergo rapid division, such as, e.g., bone marrow cells, gastrointestinal cells, hair cells, etc., and is thus associated with direct side effects [5]. Radiotherapy procedures are frequently necessary, damaging both the neoplastic focus as well as the healthy cells [6]. The use of ionizing radiation, cytostatic agents, and other immunosuppressive drugs in the course of therapy causes myelosuppression, which manifests itself in the decrease of the platelet count (thrombocytopenia), decrease of the white blood cell count (leukopenia, neutropenia), anemia and decrease of the red blood count (erythropenia) [7]. A decrease inhemoglobin content below 7g/dl is an indication for the transfusion of blood products since it constitutes a direct risk to life [8]. Significant abnormalities in blood morphology, which are an indication for transfusion, are considered by many researchers to be a marker of a severe course of CDI [9]. The period after chemotherapy where the lowest number of blood cells is present (the nadir period) constitutes a significant risk of severe complications and infections. In order to establish the magnitude of the risk, the level of neutropenia is assessed using the absolute neutrophil count (ANC) in a peripheral blood smear. The criteria proposed in NCI-CTCAE (National Cancer Institute-Common Terminology Criteria for Adverse Events), version 5.0:

Grade 1—2.0 × 10^9^/L to 1.5 × 10^9^/L
Grade 2—1.5 × 10^9^/L to 1.0 × 10^9^/L
Grade 3—1.0 × 10^9^/L to 0.5 × 10^9^/L
Grade 4—<0.5 × 10^9^/L

The risk increases with the duration of neutropenia [10,11]. The highest probability of the occurrence of infection occurs in grade 4, lasting for more than 7 days [12]. An intensifying, sudden fall of the neutrophil count within 48 h below a critical point of (500/µL = 0.5 × 10^9^/L) with a simultaneous increase of the body temperature above 38.3 °C leads to the occurrence of a neutropenic fever [13]. This condition has a negative impact on the final outcomes of oncological therapy and is characterized by high mortality; therefore, even its suspicion requires the implementation of empirical antibiotic therapy based on wide-spectrum antibiotics [14]. The lowering of the host’s humoral response potential along with antibiotic-associated dysbiosis create opportune conditions for the development of a CDI [15]. Additionally, in the case of aggressive tumors the planned treatment sequence is spread over time and may last many months, and in the case of progression, even years, which translates into repeated hospitalizations and additional medical procedures with varying degrees of invasiveness [16]. Cytostatic medications damage enterochromaffin cells of the stomach and intestines, which secrete serotonin and activate CNS receptors, causing nausea and vomiting, which requires pharmacological prevention. In the case of the parallel presence of a CD infection, the gastrointestinal symptoms may be erroneously mistaken for the action of chemotherapy, and viceversa [17]. In pediatric patients, in particular those under 2 years of age, it is necessary to commence enteral feeding through a tube [18]. The tube feeding process may be associated with complications, presenting with stomach pains, distension with gas, or diarrhea as a result of an incorrectly selected feeding substance/dose or as a result of incorrect preparation of food, causing the introduction of an infectious biological agent into the lumen of the gastrointestinal tract. It should be emphasized that the hands of medical personnel are one of the main sources of transmission of CD spores [19,20]. The use of proton pump inhibitors, which neutralize stomach acid, and non-steroidal anti-inflammatory drugs, which cause damage to the mucous membrane of the stomach during the treatment, additionally facilitates the process of infection or colonization of the gastrointestinal tract by both *Clostridioides difficile* strains and by other pathogens present in the hospital environment [21]. Surgical procedures, such as the removal of solid tumors in pediatric oncological patients, require the use of appropriate premedication, transfusion, and catheterization, and are also related to extended hospitalization and the administration of antibiotic therapy after the procedure, thus disrupting the natural intestinal microbiota [22]. The multiplicity of determinants that impact the risk of the occurrence of CDI in patients undergoing oncological treatment demonstrates that the correct diagnosis of infectious diarrhea in this group of patients is very important. In addition to identifying the patient, it also enables the implementation of appropriate procedures related to the isolation of the patient and reduction of potential transmission of the pathogen within the ward, as well as the process of controlling cleanliness and disinfection of rooms, significantly improving hospitalization safety and reducing the risk of an outbreak [23,24]. Administration of a treatment, in particular in children under the age of 2, in addition to a positive microbiological test for a toxigenic variant of CD, must be preceded by the exclusion of other factors that can cause diarrhea and must be supported by clinical symptoms. A recommendation for the collection of material for microbiological testing is prolonged diarrhea of unknown origin, usually preceded by antibiotic therapy [25,26]. In the case of children with severe disorders of peristalsis that lead to constipation and obstructions, CDI may have a non-standard course, and thus special care should be paid to the possibility of the occurrence of a toxic megacolon [27].

The objective of the study was a clinical analysis of cases related to *Clostridioides difficile* infections at a pediatric oncology department in 2020, looking at the administered treatment, the clinical parameters accompanying the disease, and the impact of the infection on the course of treatment of the primary disease.

## 2. Materials and Methods

### 2.1. General Data

The studies were conducted in a pediatric hospital qualified as a 3rd reference level, located in central Poland. The results obtained in 2020 (time range of 1 January 2020 to 31 December 2020) from patients hospitalized at an oncology department were subjected to a retrospective analysis for toxigenic variants of CD. On the basis of the collected medical documentation, an assessment of risk factors related to the CDI and of the clinical state of the patients was performed, taking into account the treatment used.

### 2.2. Laboratory Diagnostics

Tests for CD were requested as part of the diagnostic and treatment process based on the clinical symptoms observed, that is, the presence of diarrhea with varying strength, vomiting, stomach pain, tenderness, fever, general weakness, and blood in the stool. Laboratory diagnostics consisted of testing the stool samples with a two-stage algorithm for the detection of toxigenic strains of *C. difficile* and immunochromatographic assays for the detection of the antigen of glutamate dehydrogenase (GDH) and TcdA/TcdB toxins. In the case of a positive result for glutamate dehydrogenase and negative tests for TcdA/TcdB toxins, the material was cultured anaerobically on agar plates, and then from the obtained positive culture, a repeated immunochromatographic assay for CD toxins was performed. Based on the results of laboratory tests, a group of patients with positive results for toxigenic CD strains was identified. This group was subjected to a detailed review using the methods of retrospective analysis.

### 2.3. Definition of CDI

CDI cases were defined as positive in the case of a clinically significant stool (3 or more loose stools within 24 h). Due to group specifics and the severe general condition of the patients, every case of confirmation or exclusion requires an individual assessment by the medical team and the infection control team. There were special difficulties related to the appropriate qualification of patients under 2 years of age. First, it was necessary to exclude other infectious and non-infectious determinants that could result in similar clinical symptoms. The cases of hospital-acquired CDI (HA-CDI) were defined when the first clinical symptoms occurred within 48 h and were related to the hospital stay. The cases of community-acquired CDI (CA-CDI) were specified for patients with symptoms that appeared directly after admission (less than 48 h) and were not related to hospital care.

### 2.4. Statistical Methods

Elements of descriptive statistics were used in data analysis. The qualitative (category) variables were described using size (n) and frequency (%). The measurable variables were described with the following basic parameters: arithmetic mean, standard deviation (SD), median, and minimum and maximum values (min. and max.). In order to test the relationships between category variables due to a low number of study subjects (n = 30), Fisher’s exact test was used for 2 × 2 contingency tables, and a chi-square ML (maximum likelihood) test was used for contingency tables larger than 2 × 2. In order to check the significance of the difference between the two studied groups, the Mann–Whitney U test was used if the criterion of normal distribution was not met.

The *p*-value of *p* < 0.05 was assumed to be statistically significant. Statistical calculations were performed using the Statistica 13.0 and Stata/IC 12.1 software.

### 2.5. Study Populations

The study group consisted of 30 patients hospitalized in 2020 at an oncology department of a pediatric hospital, in whom positive results were obtained for toxigenic strains of *Clostridioides difficile*. The oldest patient was 15 years old, and the youngest was 5 months old. Patients under 2 years of age constituted 53.3%, whereas patients above 2 years of age constituted 46.7%. In the studied group, 66.7% were male (20) and 33.3% were female (10).

In Table 1, the original diagnosis, which constitutes the reason for oncological treatment, is presented.

## 3. Results

**Figure 1:**
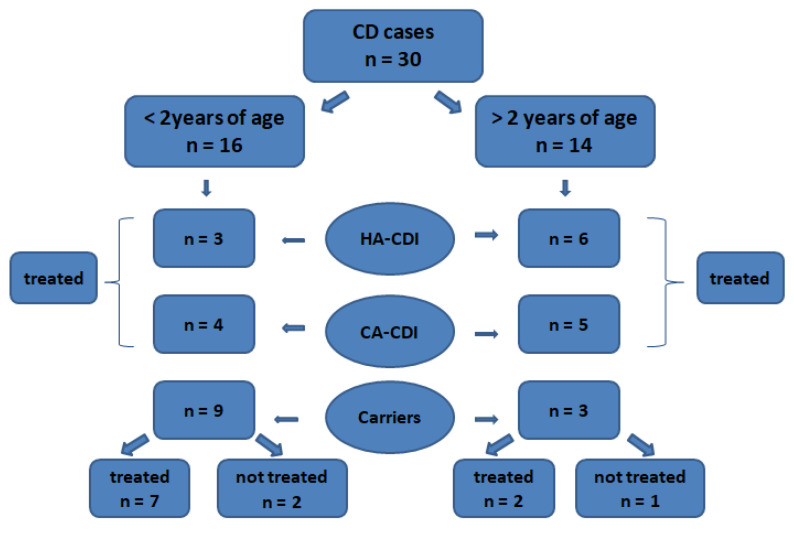
Distribution of cases related to the detection of CD, taking into account the implementation of treatment for CDI.

In 2020, the total number of hospitalizations at the oncology unit amounted to 1826, which provides an average occupancy of 51.62%, with the number of beds being 57. The average hospitalization time for the entire hospital unit, not including single-day stays, was around 3.6 days. The number of microbiological tests for CD performed during the entire year 2020 was 175 samples from 79 patients. During 12 months, a total of 37 positive results were obtained in a group of 30 patients. In 14 patients, it was the first and only diagnostic test for CD. In this group of 30 oncological patients, repeated positive results were obtained in 2 children in a period of over 4 weeks from the last test and in 2 subsequent patients in a time interval not exceeding 3 weeks from the previous test. Based on the obtained results in 9 patients, the infection was classified as a hospital-acquired *Clostridioides difficile* infection, whereas cases of community-acquired CDI were established in 9 patients as well, as reported by the Hospital Infections Control Team. Children below 2 years of age constituted, respectively, 3 cases of hospital-acquired infection and 4 cases of community-acquired infection with CDI. A group of 12 patients was classified as colonized based on a more comprehensive clinical analysis. Children below 2 years of age constituted 75% of this group. In this group, the detection of a toxigenic strain of CD was not considered to be the main determinant responsible for the gastrointestinal ailments due to the presence of other influencing factors. The main exclusion factors included, among others, a rotavirus infection, a norovirus infection, food intolerances, and primarily states after the administration of chemotherapy that cause disorders of reabsorption of fluids in the intestines and damage to the surface of the epithelium. The studied group over 2 years of age with a positive result for toxigenic CD constituted 14 patients, out of which 9 were male. Based on clinical symptoms and microbiological results, a diagnosis of hospital-acquired CDI was made in six patients. Colonization was established in 3 patients, whereas community-acquired CDI occurred in 5 patients. Figure 1 presents the qualification of cases related to the detection of a toxigenic CD strain. All confirmed cases of HA-CDI and CA-CDI were subjected to appropriate treatment.

### 3.1. Hospitalizations

**Figure 2:**
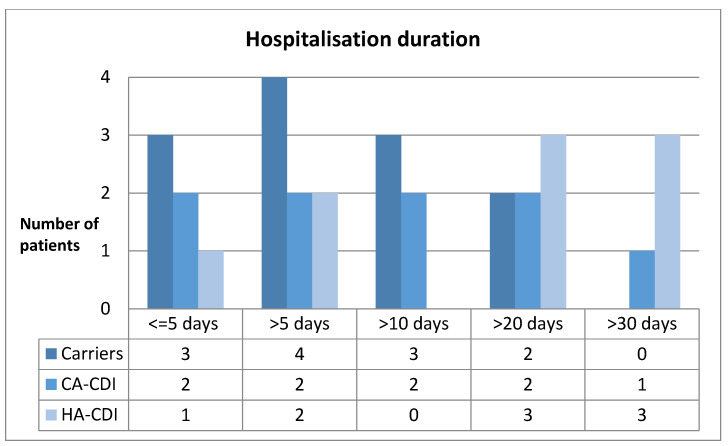
Duration of hospitalizations related to the detection of a toxigenic CD variant.

The number of hospitalizations in the entire studied group at the turn of 12 months amounted to, on average, 11.5. In the case of patients with confirmed CDI, this value was in the range of 1 to 22 (on average 10.6), whereas in the second group, the number of hospitalizations was from 4 to 22 (on average 12.9). The average duration of hospitalization was, respectively, 24.77 days (for HA-CDI patients), 16.77 days (for CA-CDI patients), and 9.75 days (for colonized patients). In 14 out of 18 cases, admissions of patients with confirmed CDI were under an emergency procedure, out of which 7 were admitted with a life-threatening condition. In 7 out of 12 cases, colonized patients were admitted to the unit in a planned manner for their scheduled treatment. The duration of hospitalization of patients admitted in a planned manner (for the entire studied group) mostly did not exceed 7 days (6 out of 11 cases). Figure 2 presents the distribution of hospitalizations resulting from a stay related to the detection of a toxigenic variant in individual groups in which positive test results were obtained.

### 3.2. Antibiotic Therapy

In the 30 days preceding the detection of a toxigenic CD variant, antibiotic treatment was administered to 25 patients. Table 2 presents the list of implemented treatments and their classification into CDI risk groups. In 11 patients, a combined therapy using more than 1 antibiotic was used (aminoglycosides were never administered in monotherapy, always in combination with a ß-lactam antibiotic). A change in antibiotic therapy occurred in seven patients and resulted from a change in the original direction of treatment. The documentation contained no mention of a change from a wide-spectrum antibiotic to one with a narrow spectrum of antimicrobial activity. Among the 5 patients who were not administered antibiotic therapy within the last 30 days, 3 were admitted to the unit in a planned manner. Among the patients with confirmed CDI, only one patient was not treated with any antibiotic, whereas as many as 8 out of 17 have been administered more than 1 antibiotic. Table 2 presents the list of used antibiotics and their classification in the CDI risk groups.

Antiviral treatment was used in 3 patients (2 colonized patients and 1 with CA-CDI). In one case, it was related to a Varicella-Zoster virus infection (a patient from the colonized group). Antifungal treatment was administered in 10 patients (7 with confirmed CDI and 3 colonized patients), with fluconazole being the active substance used in 6 patients.

### 3.3. Treatment of CDI

In the case of patients with a confirmed CDI, for 11 of them, the first-line drug was metronidazole. It was also the drug most frequently used in the group of colonized patients (5 out of 12 children). In the case of the remaining colonized patients, 4 were administered rifaximin (2 cases described above), and no CD treatment was administered to 3 of them (in one patient, symptoms resolved after the antibiotic was withdrawn, in another, after cytostatic medication was withdrawn). Vancomycin was used solely in the group of patients with confirmed CDI—originally 3 patients—and in 2 children, it was necessary to change the metronidazole used in the first-line treatment to vancomycin (patients with a relapse of the CDI).

The first case of administration of rifaximin for CD colonization was in a patient who was being prepared for megachemotherapy before a planned auto-HSCT (autologous haematopoietic stem cell transplant) procedure. The child was admitted for an emergency procedure with thrombocytopenia and symptoms of bleeding diathesis after a recent surgical procedure (enucleation of the eyeball). The neutrophil count at hospital admission was 0.14 × 10^9^ (high risk of complications). After clotting was stabilized, the therapy was continued until the patient was transferred for a transplant. The second patient was an 8-month-old infant with a diagnosis of neuroblastoma, ANC = 170/µL (cefotaxime administered at admission, blood transfusion was necessary, CRP = 30.0 mg/dl), in whom a norovirus infection was detected, a 14-day rifaximin treatment was administered, and the patient exhibited severe malnutrition (Cole index was 62.4% at BMI = 10.74).

When analyzing patients in whom treatment for CD was not administered, it should be indicated that in one patient the symptoms resolved after the antibiotic was withdrawn and in another after cytostatic medication was withdrawn (children below 2 years of age). For children above 2 years of age, only one patient colonized by tox CD was not subjected to treatment. It was a 5-year-old girl with a diagnosed malignant cerebellum cancer, admitted for another cycle of supportive chemotherapy after a recent zoster treated with acyclovir (the patient, as the only one in this age group, had not been administered antibiotic therapy previously). In the case of two patients identified as carriers, clinical data included in the medical history did not indicate the need for treatment for CD (admitted routinely, stable condition, no indications for transfusion). In addition, information was provided that diarrhea occurred immediately after the administration of chemotherapy.

### 3.4. Hematological and Biochemical Parameters

**Figure 3:**
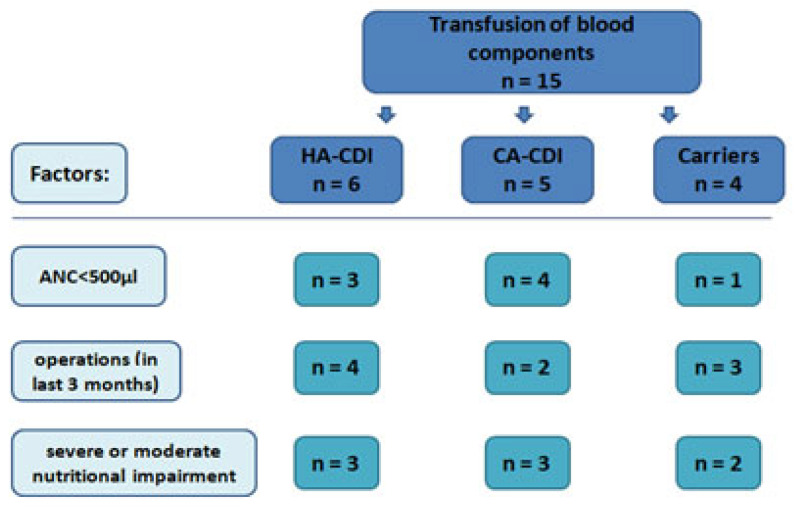
Transfusions of blood components in patients from individual groups related to the detection of a toxigenic strain of CD.

In the analyzed group, 15 patients required transfusions of blood components, with as many as 11 patients diagnosed with CDI (7 patients admitted in an emergency procedure). In the colonized group, the transfusion of blood components during an ongoing hospitalization was necessary in 4 patients, out of which in 1 the absolute neutrophil count did not exceed 500/µL (high risk of a neutropenic fever) and in one it was significantly lowered and did not exceed a level of 1000/µL. A total of 8 patients were admitted for an emergency procedure, and as much as half of this group required transfusion of blood components (in 3 patients, a hospital-acquired CDI was diagnosed, out of which in 2 patients, the time of hospitalization was significantly prolonged and amounted to 23 and 43 days). In seven patients with confirmed CDI, a high risk of the occurrence of neutropenic fever was present. The C-Reactive Protein was within limits in 11 patients (5 colonized patients and 6 children with CDI). A significant increase in this parameter was noted in five patients. Hypoproteinemia requiring the administration of preparations of albumin occurred in 7 patients, out of whom as many as 5 had CDI. Figure 3 presents the distribution related to the transfusion of blood components in individual groups of patients and additional factors that may have an impact on the state of homoeostasis.

### 3.5. Nutrition Indicators

Table 3 presents results related to the statistical analysis of the assessment of the nutritional state of colonized patients and patients with confirmed CDI. An assessment of nutritional status has demonstrated that a condition of moderate or severe impairment of nutrition occurred in eight patients. A Cole index below 75% was found in three patients. The first patient (a boy of 8 months) was classified as colonized with CD and was admitted to the hospital with severe gastrointestinal symptoms (vomiting, diarrhea, stomach pain, and fever). The diagnostic testing has indicated a norovirus infection. The patient required the transfusion of blood components due to symptoms of pancytopenia (a platelet concentrate and a leukoreduced red blood cell concentrate were transfused). Feeding was carried out with a Broviac catheter. The patient was hospitalized 18 times in 2020. Another patient (a girl aged 5) was admitted for an emergency procedure and classified as a case of CA-CDI (treated with rifaximin). Ischemia and complications resulting from the administered chemotherapy (myelosuppression and general cachexia) were found. The girl had a high risk of neutropenic fever (ANC = 300/µL), so amoxicillin with clavulanic acid was administered. After administration, ecchymosis on the skin and cold sores on the lips were observed. The patient also required the transfusion of blood components and the commencement of acyclovir treatment. Feeding was carried out through an intestinal probe. A third patient with a Cole index of 73.78% was a 9-year-old boy. After admission, ischemia, pancytopenia, and disorders related to deficiency of vasopressin (diabetes insipidus) were found. Due to the risk of neutropenic fever, amoxicillin with clavulanic acid was administered. Blood concentrate was transfused. On day 3, gastrointestinal symptoms have appeared (diarrhoea, vomiting, fever, increase in CRP). Microbiological examinations have found a toxigenic strain of CD. Treatment with metronidazole was started; the initially administered antibiotic was changed to ciprofloxacin, and fluconazole was administered. The patient was fed through an intestinal probe.

Two patients with HA-CDI were overweight: a boy of 5 with a lymphoblastic lymphoma, in whom a post-steroid Cushing’s syndrome occurred as a result of treatment (without assisted feeding), and a boy of 2 with a CNS cancer (administered dexamethasone and anti-epileptic drugs; patient fed through a probe).

### 3.6. Invasive Procedures

In nine children, invasive surgeries were performed in the last 3 months (including, among others, enucleation of the eyeball, relaparotomy, nephrectomy, hemihepatectomy, and resection of tumorsat various locations). In this group, six children had a full-blown CDI infection. In the case of one of the HA-CDI patients, bowel obstruction has occurred (distended colon on X-ray, fecal impaction without perforation, and slow peristalsis), which also required a surgical intervention. The patients being administered chemotherapy (8 patients with HA-CDI) all had a surgically implanted central venous access device. In the entire group, trepanobiopsy was conducted on five patients. Radiotherapy related to current hospitalization was conducted in five patients, four of whom were in the colonized group.

### 3.7. Hospital-Acquired Infection Charts

In the study conducted, nine patients were qualified as having HA-CDI based on the time at which the clinical symptoms occurred. In every patient, clinically significant diarrhea has occurred (in 3 patients, vomiting was additionally present). The course of the CDI in 7 patients was established as mild, whereas in 2 it was moderate. In one patient, a previous transplant was noted as a significant risk factor. Exogenous infection was established in one patient (contact infection). Table 4 contains data for patients with confirmed HA-CDI.

## 4. Discussion

The presented patient population is burdened by a series of factors that facilitate the infiltration of the pathogen and then the development of the infection. The literature data indicate that the risk of the occurrence of CDI in pediatric patients treated at oncological units is as many as 15 times higher compared to the general pediatric population, whereas the risk of the occurrence of CDI may apply even to 17% of children who receive HSCT [28,29,30]. The high level of CD colonization present in infants and children below 2 years of age is an additional risk factor for the remaining patients at the unit and leads to contamination of the hospital environment. This concerns colonization by toxigenic strains, which may be frequently acquired as a result of multiple hospitalizations. Rzayev et al. (2021), in a study of CD colonization in children before and after hospitalization, noted that in a group of 49 patients admitted colonized with non-toxigenic CD strains, at the end of hospitalization with antibiotic therapy, toxigenic CD was detected in 6% [31]. It should be emphasized that CD colonization in standard cases does not require treatment and should be subjected to a thorough differential diagnosis in symptomatic cases of CDI. In routine hospital practice, tests for detecting colonization with toxigenic strains of CD in patients admitted to the department are not conducted. This significantly hinders the assessment of the epidemiological situation and creates a transmission pathway [32]. In the study of 14 patients, it was the first and only test for CD performed in 2020. CD as a factor of full-blown infection was confirmed in 18 patients, while the treatment was administered in as many as 27 patients, that is, also in children defined as carriers. In two cases, the documentation did not provide clear premises on the basis of which treatment was initiated in carriers. The occurrence of an episode related to gastrointestinal disorders (diarrhoea, vomiting, or persistent stomach pain) every time impacted decisions concerning the implemented plan of cancer treatment, and in 86% of cases, it was associated with postponing the planned cycle of chemo- and/or radiotherapy for a few days. The medical history charts did not note a direct impact of postponing the implementation of treatment on the process of oncological therapy. However, a highly disciplined oncological therapy is related to a rigorous schedule of chemotherapy, and deviations may, from a long-term perspective, impact the period of remission and the time free from progression of disease. The detection of a toxigenic variant of CD and a decision on treatment made by a doctor were related to an extension of the hospitalization process (an average of 10.3 days), resulting in additional treatment costs. In the case of a patient classified for HSCT therapy, the eradication of CD was first necessary. This has caused an extension of the procedure’s waiting time.

Whereas in the case of a patient with end-stage neoplastic disease, the course of a CD infection has negatively impacted the general condition of the patient despite the use of palliative therapy intended to increase the comfort of life. In the discussed case, it is difficult to unequivocally consider CD to be a factor impacting shorter survival due to the progression of the disease.

In a study we conducted previously, an analysis of the state of the hospital environment at a pediatric oncology unit demonstrated the presence of CD spores on elements in direct contact with patients and on parts of doctors clothing. The study was performed using C. diff Banana Broth™ medium (Hardy Diagnostics, Santa Maria, USA), which is used for culturing and recovering CD spores and vegetative cells from environmental samples. The obtained results indicate the main sources of spore transmission and the need for continuous monitoring of the situation of high-risk patients, in whom long-term contact with the hospital environment is a very significant prognostic factor for the occurrence of a CDI [33]. In this perspective, nutrition disorders and the use of assisted feeding may open the gates for the colonization of the gastrointestinal tract due to the difficulties of maintaining strict aseptic conditions. In our study, CDI recurred in 2 patients, whereas as many as 8 children have obtained a negative result in a previous test for CD. Acquisition of the pathogen from the hospital environment seems the most probable scenario, in particular in the case of multiple hospitalizations, which are always necessary in oncology treatment.

The obtained results indicate that as many as 17 patients with a confirmed CDI infection have been administered antibiotics in the last 2 weeks, out of which 88% were antibiotics related to a high risk of a CD infection (amoxicillin, clindamycin, and fluoroquinolones). Wide-spectrum antibacterial antibiotic therapy could have been the main factor determining the occurrence of CDI symptoms. In most cases, empirical treatment started after admission to the hospital was not replaced with targeted treatment. A wide bactericidal spectrum of the listed drugs results in disturbances to the natural microbiota, the selection of resistant strains, and conditions favoring the germination of CD spores. A meta-analysis by Brown and a previous study by Bartlett indicate different clinical risks of CDI occurrence for different antibiotics. Clindamycin, cephalosporins, and fluoroquinolones may constitute the greatest hazards [34,35]. In the study we have conducted, the antibiotic with the most probable correlation with CDI was amoxicillin with clavulanic acid. Additionally, our studies indicate a high exposure to wide-spectrum antibiotics both in the group of confirmed infections and in the group of asymptomatic carriers. Oncological patients are frequently administered antibiotic therapy, including antivirals and antifungals, due to their clinical state. Only one patient from the group with a confirmed CDI infection in the last 3 months was not administered an antibiotic, whereas the entire group was subjected to treatment directed against CD. It should be noted that in the case of colonized patients, treatment for CD was administered to as many as ¾ patients. Only in three children was it decided to stop the administration of antibiotics and postpone chemotherapy, which, according to the information in the medical history, had positive outcomes in the form of the end of gastrointestinal aliments. In most cases, the drug used in first line treatment of CDI was metronidazole. Vancomycin was administered in cases of recurrence and in patients with more severe symptoms. The use of oral vancomycin selects for VRE (Vancomycin Resistant Enterococci)strains, so minimizing its use is very important in order to limit hospital-acquired infections with highly resistant strains. Due to the series of other risk factors, every decision required a more comprehensive clinical analysis. We have noticed that most of our patients had a terminal or uncertain prognosis, whereas the diagnosed cancers were located mainly in the CNS (central nervous system). Exposure to medication used in chemotherapy regimens could also, in this case, be related to a higher probability of CDI. Abughanimeh (2018) indicates that a mechanism by which chemotherapy causes an increase in CDI risk has never been established. However, these medications have a significant impact on intestinal membranes, impacting their ability to regenerate and damaging mucous membranes. The occurrence of CDI in the case of chemotherapeutic agents may be detected without the use of antibiotic therapy [36]. The researchers have noted that the administration of chemotherapy regimens that result in injuries to the gastrointestinal tract has been related to extended immunosuppression, frequent and extended hospitalization, and increased use of antibiotics. Hematological patients may be an example (with a risk of CDI 2.5 times higher compared to solid tumors). We have noticed that the length of hospitalization in oncological patients with confirmed CDI was on average 5 days longer compared to the colonized group; additionally, they were admitted mainly for an emergency procedure. Being admitted to the department in a life-threatening situation or requiring urgent medical intervention was related to the implementation of an appropriate therapy. In our group, 73.3% of CDI patients required the transfusion of blood components, and in seven, a high risk of neutropenic fever occurred. Immune response disorders related to low neutrophil counts required the introduction of antibiotic therapy.

The use of general anesthesia and opiates may impair the functions of the gastrointestinal tract. In mice exposed to morphine, researchers have shown changes in intestinal epithelium and suppression of intestinal mucus [37]. In our study, every thirdperson in the last half year was subjected to a surgical intervention, which, in the situation of impairment of the immune system and the presence of additional factors, could have played a role in the pathogenesis of CDI.

### Limitations

Our study had some significant limitations, mainly related to the COVID-19 pandemic. Due to the specifics of CD infections in pediatric population, there are difficulties in collecting a wider group at a single medical facility. The data contained in the documentation of patients often omitted significant data concerning changes in disease dynamics or attachments resulting from referral to diagnostic examinations. It should be emphasized that it was a single-center study, focusing on a single unit, with the specifics and high complexity of cases and risk factors preventing the comparison of this group of children to other patients. Our observations indicate that the course of an oncological disease may differ completely even in the case of the same type of cancer; therefore, significant heterogeneity exists within a single group. In Poland, the total number of all reported cases of CDI in children and teenagers in 2020 was 307 (in 16 provinces). In the Chief Sanitary Inspectorate reports, there is no information concerning what percentage of these are oncological patients. The dynamics of the spread of infections caused by CD require strict epidemiological control. Establishing the clinical conditions related to infections caused by CD in pediatric oncological patients becomes more and more important in the context of the medical consequences and risks resulting from the delaying of the primary treatment.

## 5. Conclusions

The conducted study demonstrates that infections caused by toxigenic CD strains constitute a real diagnostic and therapeutic problem in pediatric patients. Antibiotic therapy is one of the main factors determining the occurrence of a CDI. A dangerous phenomenon was the lack of a noticeable change from the primary antibiotic therapy to drugs acting on a narrower antibacterial spectrum. The highest risk of CDI was associated with the use of beta-lactam antibiotics (with a broad spectrum of antibacterial activity), such as amoxicillin with clavulanic acid and third-generation cephalosporins. The possibility of reducing or substituting the use of the drug should be assessed. Cases of treatment for CD without reasonable grounds should be excluded. Oncological treatment combined with additional risk factors, such as malnutrition, low neutrophil count (high risk of neutropenic fever), transfusion of blood components, assisted feeding, and protein disturbances, may, in our opinion, constitute additional CDI-related prognostic factors. Medical interventions undertaken in the process of treating an oncological disease generate consequences that may impact the occurrence of CDI. Excluding all coincidences and making unequivocal recommendations, in our opinion, requires a multi-centerstudy at pediatric oncology units.

## Figures and Tables

**Table 1:** The original diagnosis in oncology patients with a detected toxigenic strain of *C. difficile*.

Original Diagnosis	Number of Patients	Patients with CDI	Patients Colonized by CD
Neuroblastoma type 4	4	2	2
Malignant neoplasms of the brain and CNS	8	3	5
Malignant neoplasms of the eye	3	1	2
Malignant neoplasms of the liver	4	3	1
Lymphoma	2	2	-
Malignant neoplasm of the kidney	2	1	1
Non-malignant neoplasm of brain and other parts of CNS	1	1	-
Neurofibromatosis type 1	1	1	-
Malignant neoplasm of connective tissue	1	1	-
Malignant neoplasm of the ovary	1	1	-
Rhabdomyosarcoma, RMS	2	1	1
Malignant neoplasm of soft tissue	1	1	-

**Table 2:** Burden related to the antibiotic therapy administered during the last 30 days in the group of patients with a positive resultfor toxigenic *C. difficile.*

Antibiotic Group	Administered Antibiotic	Number of Patients Subjected to Treatment	Classification of CDI Risk
3rd generation cephalosporins	Ceftriaxone	8	High risk
	Cefotaxime	5	
	Ceftazidime	2	
Beta-lactam antibiotics with an inhibitor	Amoxicillin with clavulanic acid	12	High risk
Aminoglycosides	Amikacin	5	Low risk
Lincosamides	Clindamycin	3	High risk
Fluoroquinolones	Ciprofloxacin	3	High risk
Sulfonamides	Trimethoprim/Sulfamethoxazole	3	Low risk
Macrolides	Clarithromycin	2	Medium risk
Carbapenems	Imipenem	1	Medium risk
	Meropenem	1	
Glycopeptides	Teicoplanin	1	Low risk

**Table 3:** Assessment of nutritional status in patients with confirmed CDI and in the group of colonized patients.

Factor		Total		Patients with Confirmed CDI		Patients Colonised with CD		*p*
		n	[%]	n	[%]	n	[%]	
Gastrointestinal tract symptoms up to 48 h since admission	No	13	43.33	9	50.00	4	33.32	0.3669 ^(1)^
	Yes	17	56.67	9	50.00	8	66.68	
Feeding	Normal	5	16.7	1	5.56	4	33.32	-
	Broviac catheter	7	23.3	5	27.8	2	16.66	
	Intestinal probe	17	56.7	11	61.10	6	50.00	
	Gastrostomy	1	3.3	1	5.56	0	0.00	
Cole index%	M ± SD Me (min.–max.)	92.9 ± 13.3 94.6 (62.4–123.7)		92.4 ± 14.3 90.9 (73.2–123.7)		93.4 ± 12.2 95.5 (62.4–105.7)		0.6114 ^(2)^
Assessment of nutritional state	Overweight	2	6.7	2	11.11	0	0	-
	Correct nutrition state	16	53.3	7	38.89	9	75.00	
	Mild impairment of nutrition state	4	13.3	3	16.67	1	8.33	
	Moderate impairment of nutrition state	5	16.7	4	22.22	1	8.33	
	Severe malnutrition	3	10.0	2	11.11	1	8.33	

Statistically significant (*p* < 0.05). M—arithmetic mean; SD—standard deviation; Me—median; min.–max.—minimum and maximum values; *p*—probability value; ^(2)^—result of the Mann–Whitney U test; ^(1)^—result of chi-square ML. Cole index [%]: 90–110%—overweight; 85–90%—mild impairment of nutrition state; 75–85%—moderate impairment of nutrition state; 75% and lower—severe malnutrition.

**Table 4:** Hospital-acquired infection charts for CDI patients.

Hospital-Acquired Infections Charts									
	Patients								
Age	2	7	3	9	1	12	2	5	3
Sex: male (M), female (F)	M	F	M	M	F	M	M	M	F
Antibiotic therapy	+	+	+	+	+	+	+	+	+
Chemotherapy	+	+	+	+	+	+	+	−	+
Immunosuppression	+	−	−	+	−	+	−	+	−
Advanced stage of disease	+	+	+	+	+	+	+	+	+
Organ transplant	−	−	−	−	−	+	−	−	−
Central venous access device	−	+	+	+	−	−	+	−	+
Extended hospitalisation	+	+	+	+	+	+	+	+	+
Another stay	+	+	+	+	+	+	+	+	+
Admitted from home	+	−	+	+	+	+	+	−	+
Admitted from another unit	−	+	−	−	−	−	−	+	−
Clinical course of infection: mild (Mi), moderate (Mo)	Mi	Mi	Mi	Mi	Mi	Mo	Mo	Mi	Mi
Treatment: metronidazole (MTZ), vancomycin (VA), rifaximin (R)	MTZ	MTZ	MTZ	MTZ	R	VA	MTZ/ VA	MTZ	MTZ
Diarrhoea	+	+	+	+	+	+	−/+	+	+
Vomiting	−	−	+	−	+	+	+	+	−
Fever	−	−	−	+	−	+	+	+	−
Implemented procedures: isolation (I), cohortation (CO)	I	I	I	CO	I	I	I	I	I
	Disinfecting the unit								
Type of infection: endogenous (EN)/exogenous(EX)	EN	EN	EX	EN	EN	EN	EN	EN	EN

## Data Availability

The data presented in this study are available upon request from the corresponding authors.
